# The Ideal Goal of Testosterone Replacement Therapy: Maintaining Testosterone Levels or Managing Symptoms?

**DOI:** 10.3390/jcm8030362

**Published:** 2019-03-14

**Authors:** Du Geon Moon, Hyun Jun Park

**Affiliations:** 1 Department of Urology, Korea University Guro Hospital, Korea University College of Medicine, Seoul 08308, Korea; uromoon@gmail.com; 2 Department of Urology and Medical Research Institute of Pusan National University Hospital, Pusan National University School of Medicine, Busan 49241, Korea

Typically, the goal of testosterone replacement therapy (TRT) is to restore blood testosterone to normal levels. When used to treat men with hypogonadism, it may also result in other benefits including (i) improved sexual desire and erectile function, (ii) improved energy, mood, and vitality, (iii) increased lean body mass, (iv) reduced waist circumference, (v) reduced total body fat mass, (vi) increased bone mineral density, (vii) increased insulin sensitivity, (viii) reduced blood glucose and hemoglobin A1c, and (ix) increased muscle strength as shown in Table 1 [1,2]. Improvement in libido generally occurs within 6 weeks of treatment, whereas other benefits usually take up to 12 months [3]. Thus, the effects of TRT are very diverse. Several clinical trials have been conducted and the effects of TRT have already been well established in published papers [4]. The questions that most practitioners prescribing TRT have are regarding the realistic therapeutic endpoints that must be set for TRT. According to the currently published guidelines, it is recommended that the concentration of testosterone is restored to the mid-normal range (International Society for Sexual Medicine) [5] or to the middle tertile of the normal reference range (American Urological Association) [1]. These recommendations, however, do not provide appropriate answers to the practitioners in real-world settings. In addition, many published studies on TRT have used different outcome measures depending on the research themes as shown in Table 1 [4]. Another problem that confuses practitioners is that serum testosterone levels and symptoms are not mechanically correlated. In some cases, the testosterone level is restored to normal without any improvement in symptoms after a sufficient period of time, whereas in other cases, testosterone levels are still low with the improvement in symptoms. Although very difficult, it is necessary to set the ideal TRT goal for practitioners who start TRT in these situations. However, it is recommended that treatment goals are established to achieve an appropriate level of testosterone (e.g., 3−5 ng/mL) rather than subjective symptom improvement. This is because the duration of treatment required for the improvement in subjective symptoms varies according to each symptom. In addition, there are different test methods that can objectively evaluate each symptom. Therefore, it is difficult to assess all the symptoms related to testosterone deficiency in real practice settings. It is important to identify the symptoms that are most obvious and uncomfortable for the patients before treatment. After treatment, it is recommended that focus is directed towards assessing whether the serum testosterone level has reached the normal range and whether symptoms have improved. If the symptoms are sexual libido or erectile dysfunction, patients are more likely to appreciate the effects of treatment on the symptoms. However, symptoms such as changes in body composition, bone mineral density, lean body mass, muscle strength, etc., are difficult for the patient to appreciate immediately and require assessment of the treatment effects and feedback of the test results. This increases the patient’s satisfaction with treatment and induces self-awareness for improvement in symptoms. If there is an improvement in the subjective symptoms after treatment without any change in serum testosterone levels, these might be placebo effects. It is therefore advisable to discontinue TRT as recommended in the guidelines [1,5]. In summary, the ideal goal of TRT is to focus on the recovery of serum testosterone levels. Regarding the improvement of subjective symptoms, it is recommended that patients are encouraged to be self-aware or provide feedback by appropriate tests.

## Figures and Tables

**Table 1:** Benefits of testosterone therapy and outcome measures.

Benefits of Testosterone Therapy	Estimated Time to Effects	Outcome Measures
Improved sexual desire and erectile function	3–6 weeks	IIEF, AMS, HADS, GEQ, PDQ, DISF/DISF-SR
Improved energy, mood and vitality	3–6 weeks	AMS, HADS, GEQ, SF-12, fatigue (MFI), PHQ-9, BDI-II, BAI
Increased lean body mass	12–16 weeks	DXA
Reduced waist circumference	12–16 weeks	Waist circumference
Reduced total body fat mass	12–16 weeks	DXA
Increased bone mineral density	6 months	DXA
Increased insulin sensitivity	Few days	HOMA
Reduced blood glucose	3–12 months	Glycated hemoglobin (HbA1c), fasting plasma glucose
Increased muscle strength	12–16 weeks	Maximum weight lift, bench and leg press exercises

IIEF, International Index of Erectile Function; AMS, Ageing Male Symptom Questionnaire; HADS, Hospital Anxiety and Depression Scale; GEQ, Global Efficacy Question; PDQ, Psychosexual Daily Questionnaire; DISF/DISF-SR, Derogatis Interview for Sexual Function; SF-12, Short-Form-12; MFI, Multidimensional Fatigue Inventory; PHQ-9, Patient Health Questionnaire-9; BDI-II, Beck Depression Inventory; BAI, Beck Anxiety Inventory; DXA, Dual energy X-ray absorptiometry; HOMA, Homeostasis model assessment index of insulin resistance.
